# Depression and Anxiety Disorders among Patients with Psoriasis: A Hospital-Based Case-Control Study

**DOI:** 10.1155/2012/381905

**Published:** 2012-07-16

**Authors:** Masoud Golpour, Seyed Hamzeh Hosseini, Mohammad Khademloo, Maryam Ghasemi, Aghdas Ebadi, Fatemeh Koohkan, Soheila Shahmohammadi

**Affiliations:** ^1^Department of Dermatology, Booali Sina Hospital, Mazandaran University of Medical Sciences, Sari, Iran; ^2^Behavioral Science and Psychiatric Research Centre, Zare Psychiatric Hospital, Mazandaran University of Medical Sciences, 5th Kilometer Sari-Neka Boulevard, Mazandaran Province, P.O. Box 48167-15793, Sari, Iran; ^3^Department of Community Medicine, Faculty of Medicine, Mazandaran University of Medical Sciences, Sari, Iran; ^4^Department of Pathology, Booali Sina Hospital, Mazandaran University of Medical Sciences, Sari, Iran; ^5^Faculty of Medicine, Mazandaran University of Medical Sciences, Sari, Iran; ^6^Nursing and Midwifery Faculty, Mazandaran University of Medical Sciences, Sari, Iran

## Abstract

*Background*. Psoriasis is a common, genetically determined inflammatory and proliferative disease of the skin. Psychological stress can exacerbate the disease. This study sought to investigate the depression and anxiety disorders among patients with psoriasis and control group. *Method*. In this hospital-based case-control study, One hundred patients with psoriasis (case) referred to the dermatology department and 100 patients with otolaryngology problems and dermatological healthy volunteers (control) who referred to the Otolaryngology Department of Bouali Sina Hospital in Sari, Iran, in 2007 were studied. Demographic characteristics were recorded. Beck Depression Inventory and Spielberger State-Trait Anxiety Scale I-II were administered to the patients in both groups. Data were analyzed using SPSS statistical software and descriptive statistical tests. *Results*. From One-hundred patients in each group, 44 (45%) were men. Depression score was 67% and 12% in psoriatic patients and control, respectively. The Beck depression scores of patients with psoriasis were significantly higher than scores of the control group (*P* < 0.05). Based on Spielberger State-Trait Anxiety Scale, anxiety was found in 45% of patients in case group and 18% of controls. *Conclusion*. The results revealed that psoriatic patients reported significantly higher degrees of depression and anxiety than controls. In addition, psoriatic women were more depressed than psoriatic men.

## 1. Introduction

Psoriasis is a skin disorder affecting approximately 1–6% (mean 3%) of populations in the world. Two-peak age of onset was considered for the disease; the early age of onset is between 16–22 years, and latent age of onset is between 57–60 years. The incidence of psoriasis in adult men and women and among different races is equal. However, females tend to develop the disease earlier than males. There are several classes of psoriasis including: psoriasis vulgaris, guttate psoriasis, generalized pustular psoriasis, disseminated erythrodermic psoriasis, scalp psoriasis, palms and soles psoriasis, nail psoriasis, arthropathic psoriasis, and verse psoriasis. It is believed that a combination of several factors contribute to the development of this disease. Genetic factors, trauma, infection, certain medicines, such as nonsteroid anti-inflammatory drugs (NSAIDs), beta-blockers, antimalaria medicine, and lithium, endocrine factors, sunlight, metabolic factors, alcohol, cigarette, and psychological factors have been found in development of psoriasis [1].

There is strongly clinical evidence that stress can play a role on the onset and exacerbation of psoriasis [2–5].  In a study on psoriatic patients, 60% of the patients strongly believed that stress was a causal factor for their psoriasis [6].

Psoriasis makes stress itself, and in turn, stress can worsen psoriasis. However, most Psoriatic patients who reported episodes of psoriasis precipitated by stress describe disease-related stress, resulting from the cosmetic disfigurement and social stigma of psoriasis [1].

Psoriasis is associated with a variety of psychological problems. So, considering the psychosocial aspects of the disease is very important in psoriatic patients [7].

 According to previous controlled studies, the prevalence of depression was ranged from 0 to 58% in psoriasis patients [3]. One study has demonstrated that female psoriatic patients appear to be more vulnerable to develop depression than males. The prevalence of anxiety is higher than depression in psoriatic patients. Even psoriatic patients have reported significantly higher degrees of anxiety than other chronic diseases such as cancers. Furthermore, the severity of anxiety would be greater in patients with palms and soles psoriasis [3].

Psoriasis is associated with a variety of personality disorders. On the other hand, psychological stress can induce resistance to regular psoriatic treatment and causes psoriasis to appear worse. In this view, psoriasis is an inflammatory disease with expensive and long-term therapies, and as mentioned before, psychosocial stress can exacerbate the disease. So that, recognition and treatment of the psychosocial problems can decrease_health care costs and shorten the therapeutic period. Therefore, we decided to compare depression and anxiety disorders in patients with psoriasis and the control group.

## 2. Materials and Methods 

In this hospital-based case-control study, all participants were patients who referred to Department of Dermatology at Booali Sina Hospital in Sari/Iran during 2007. The ethics committee of Mazandaran University of Medical Sciences approved the study protocol. Written informed consent was obtained from all participants. Diagnosis of psoriasis was based on clinical examination by a dermatologist and confirmed by histological examination of the lesions by a pathologist. The control group consisted of 100 patients referred to the Otolaryngology Department of the hospital with no dermatology problems at the same time.

The exclusion criteria were as follows:

1. other dermatologic diseases,
2. individuals with mental retardation or cognitive and speech disorders.

Each group were matched by variables including age and sex. A dermatologist confirmed the diagnosis of psoriasis based on observations and clinical findings. Biopsy was taken from suspected lesions and sent to the pathology center of the hospital. For the patients whose samples were positive after H&E stain and microscopic examination (hyperkeratosis, severe parakeratosis, hypogranulosis or absence of granular layer, suprapapillary epidermal thinning, dilated tortuous papillary capillaries, microscopic collection of polymorphonuclear white blood cells in stratum corneum layer (Munro abscess) or in the malpighian layer (Kogoj abscess), pericapillary infiltration of lymphocyte and neutrophils) appropriate treatment was started.

In addition, a dermatologist examined the control group for presence of dermatological diseases. Demographic characteristics such as sex, age, job, level of education, type and site of lesions, and extension of the disease were recorded. Then the patients in both case and control groups were evaluated by the Beck Depression Inventory (BDI) and Spielberger State-Trait Anxiety Scale (STAI I-II) questionnaires.

Suspected cases with depression or anxiety based on BDI and STAI I-II tests were referred to a psychiatrist for clinical diagnosis. The psychiatrist performed semi structural clinical interview according to DSM IV-TR to determine existence and/or type of disorder.

The Beck Depression Inventory (BDI) is a 21-item self-reporting scale developed to measure the severity of depression. Each item gets scores ranging from 0–3. The score for depression can vary from a minimum of 0 to a maximum of 63. In this study, the BDI score of more than 17 was considered as presence of depression and referred to the psychiatrist. Then clinical interview was performed to identify type of psychiatry disorders based on DSM IV. Content validity of BDI has been extensively tested and confirmed by previous studies [8].

The State-Trait Anxiety Inventory (STAI) is a self-report assessment device, which includes separate measures of state and trait anxiety. It is a 40-item scale made up of two 20-item subscales (one state and one trait) to assess the anxiety.

The state anxiety scale describes the individual's feelings at a particular time and under particular conditions, whereas the trait anxiety scale describes the usual feelings of the individual.

Each STAI item is given a weighted score of 1 to 4. A rating of 4 indicates the presence of high levels of anxiety for some items, and the scoring weights for the anxiety-absent items are reversed. Scores for both the S-anxiety and the T-anxiety scales can vary from a minimum of 20 to a maximum of 80.

The score >43 was considered as presence of anxiety and was referred to the psychiatrist. To identify type of psychiatric disorders, clinical interview was done using DSMA IV. Content validity of STAI has been confirmed by previous studies [9].

Known Psoriatic patients were followed and they received proper treatment. All analyses were performed using SPSS software and *χ* ^2^ tests.

## 3. Results

There were 100 patients (44 males (45%) and 56 females (55%)) in the case and control groups. Their age range was between 20 and 50 with a mean of 34.28 ± 15.50 and 34.45 ± 14.4 in case and control, respectively. In the control group, 5.2% were at elementary-junior high school education level, 9.3% were in grade high school, 24.7% were diploma, and 60.8% had university education level. The education levels in the case group were as follows: elementary-junior high school 23.5%, high school 15.3%, diploma and university education level were 34.7% and 26.5%, respectively.

There was no evidence for chronic diseases such as hypertension, diabetes, asthma, and rheumatic disorders in history of the patients in the case and control groups. There was no history of drug users in either of the studied groups.

Ninety-nine (99%) were on topical therapy while the other one (1%) were receiving systemic as well as topical therapy.

Ninety-five (95%) were plaque psoriasis (psoriasis vulgaris), three (3%) guttate [GUH-tate] psoriasis, one (1%) inverse psoriasis, and one (1%) pustular psoriasis. There was no erythrodermic psoriasis or psoriasis arthritis. Duration of the disease was less than one year among all the psoriatic patients.

**Figure 1:**
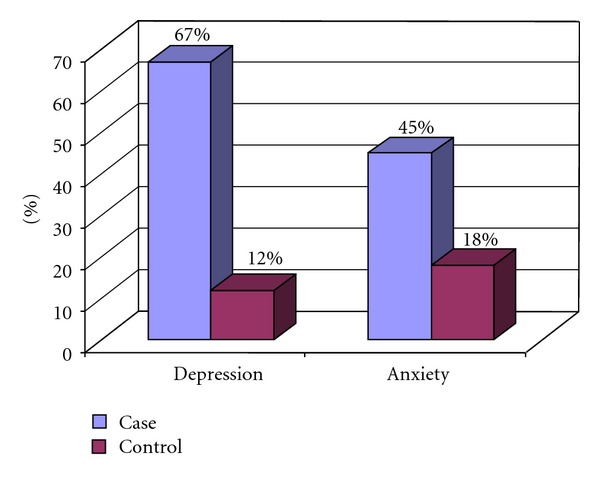
Comparison of depression and anxiety levels between the case and control groups.

The Spielberger questionnaires indicated that 45% and 18% of case and control groups had anxiety, respectively (Figure 1). This finding was statistically significant (*P* < 0.001). Psoriatic patients showed higher level of anxiety than the control group. There was no relationship between anxiety and sex, education level, and employment in the case group, and we found no significant statistical differences (Table 1).

The Beck Depression questionnaires indicated that 67% and 12% of the patients in the case and control groups had depression, respectively. This result demonstrated significant differences between both case and control groups (*P* < 0.001J) (Figure 1).

The case group was assessed for relationship between depression and sex, education level, and employment, but there was no statistical significant difference (*P* > 0.05).

In the case group, depression was found in 11 of employer's patients, 11 school or university students, 9 homemakers, and 2 unemployed. This finding was also significant statistically (*P* = 0.008).

Excluding demographic characteristics such as age, sex, job, and education, the risk of psoriatic disease related to anxiety and depression would have been 4.6 times (2.11–11.7) and 6 times (2.37–17.41) of healthy subjects, respectively.

According to semi-structural interview by the psychiatrist, psychiatry disorders included 43 mixed anxiety and depression disorder (43%), 9 mild major depression disorder (9%), 7 adjustment disease (7%), 4 social phobia (4%), and 37 no mental disorder (37%) in the case group, but 8 mixed anxiety depression (8%), 4 adjustment disease, and 88 no mental disorder were found in the control group which signifies the difference between the two groups (*P* < 0/001).

## 4. Discussion

According to Beck Depression Inventory (BDI) and Spielberger State-Trait Anxiety Scale (STAI), psoriatic patients had higher degrees of depression and anxiety than normal subjects. In addition, Psoriatic women reported higher degrees of depression than psoriatic men.That is similar to the results of Akay et al.[10].

Naldi et al. [11], in a case-control study, found that family history of psoriasis, stressful life events, and recent infectious diseases (like streptococcal pharyngitis group B) are risk factors for a first episode of guttate Psoriasis; the results of our study confirm this finding.

The role of stressful life events in the development of guttate Psoriasis is estimated 1.7% (0.8–3.6) and an increased comorbidity with pharyngeal infection was found in patients with guttate Psoriasis, whereas our results revealed that comorbidity with anxiety and different kinds of Psoriasis is 4.6% (2.11–11.7).

Sampogna et al. in 2006 have studied the effects of age, gender, quality of life, and psychological distress in 936 hospitalized patients with psoriasis. They divided patients into two age groups: younger than 65 years, and older than 65 years. They found that the older psoriatic patients had lower quality of life and they were exposed to more stressful life events. In addition, women reported more depression than men [1, 12]. 

In another study by Zachariae et al., on quality of life (QOL) in 6497 Nordic patients with psoriasis, psoriatic women had lower QOL than psoriatic men [13].

Similar to ours, O'Leary et al.'s study showed that the magnitude of anxiety and depression in patients with psoriasis is higher than that in healthy individuals, but they found no association between severities of psoriasis with anxiety [14], whereas Harvima et al. found an association between psychological stress and clinical severity and symptoms in psoriatic patients. They had found patients with significant psychological distress who had more severe dermatologic lesions and particular defects. The sample size in O'Leary and Harvima studies was 141 and 38 patients, respectively. Therefore, the sample size may lead into different results [13, 15].

Taner et al. in 2007 compared the rate of depression and anxiety in patients with Behcet's disease and in patients with psoriasis. The results of their study revealed that the prevalence of anxiety in young patients with Behcet's disease was higher than that in the psoriatic patients [16]. Furthermore, there was a positive correlation between the severity of psychological symptoms and longer duration of the disease in patients with Behcet's disease. However, there was no correlation between the duration of illness and psychological symptoms in patients with psoriasis. Our study had two unique aspects.

1. Although all previous studies believed that anxiety and depression have been effective on incidence of psoriasis, in our study, the risk of developing psoriasis at presence of anxiety and depression was investigated regardless to age, sex, profession, and education level of the patients.
2. A clinical interview was performed to identify the type of psychiatry disorders based on DSM IV. In addition, we investigated the correlation between job and education level on incidence of depression and anxiety in the psoriatic patients. Though there was no correlation between the education level and incidence of depression and anxiety, lower depression levels were observed in employed patients.

There were some limitations in our study. For example, the profession and education levels were interventional variables that were not matched between the two studied groups. Besides, the study only included the new cases of psoriatic patients with depression and anxiety who referred to the psychiatrist after completing the questionnaire. In addition, psychiatry problems such as schizophrenia, MDD, and personality disorders in subjects with mental retardation, cognitive and speech disorders as exclusion criteria in the present study had not been diagnosed after clinical interview.

## Figures and Tables

**Table 1:** Demographic variables based on anxiety and depression in psoriatic patients.

	Depression					Anxiety				
Variables	Yes		No		*P* value	Yes		No		*P* value
	no	%	no	%		no	%	no	%	
Sex										
Female	24	21.4	88	78.6	0.68	36	32.1	76	67.9	0.82
Male	21	23.9	67	76.1		27	30.7	61	69.3	

Education level										
Elementary—junior high school	7	25	21	75		11	39.3	17	60.7	
High school	8	33.3	16	66.7	0.53	5	20.8	19	79.2	0.47
Diploma	11	19	47	81		16	27.6	42	72.4	
University	18	21.2	67	78.8		28	32.9	57	67.1	

Profession										
Employment	18	18.8	78	81.3		28	29.2	68	70.8	
School-university student	14	38.9	22	61.1	0.7	12	33.3	24	66.7	0.6
Housewife	11	21.6	40	78.4		19	37.3	32	62.7	
Unemployment	2	14.3	12	85.6		3	21.4	11	78.6	
